# Brewing Anxiety: Caffeine‐Induced Thigmotaxis in Zebrafish Larvae

**DOI:** 10.1002/brb3.71769

**Published:** 2026-09-30

**Authors:** Rishi Rai, Thais Del Rosario Hernandez, Robbert Creton

**Affiliations:** ^1^ Department of Molecular Biology, Cell Biology and Biochemistry Brown University Providence Rhode Island USA

**Keywords:** anxiety, high‐throughput screening, thigmotaxis, zebrafish

## Abstract

**Background:**

Thigmotaxis, or edge preference, is widely used as a measure of anxiety in vertebrate model systems. However, variability in this behavior limits its utility as a scalable measure for anxiolytic drug discovery. In the current study, we examine caffeine‐induced thigmotaxis, using 5‐day‐old zebrafish larvae as a model system.

**Method:**

We modified our imaging system and AI‐assisted analysis for measurements of behavior in six‐well plates. The imaging system was used to measure thigmotaxis and several other behaviors, including overall activity, scoot and burst swimming, and responses to acoustic and visual stimuli.

**Result:**

Caffeine (100 mg/L) caused the zebrafish larvae to exhibit increased edge preference, decreased activity, increased scooting, and decreased bursting behavior. Coadministration with diazepam (0.25 mg/L), a central nervous system depressant, significantly reduced caffeine‐induced thigmotaxis and increased locomotor activity.

**Conclusion:**

These findings support edge preference as a reliable measure of anxiety and reconcile existing conflicting results regarding diazepam's anxiolytic efficacy in zebrafish larvae, suggesting that anxiolytic effects are most pronounced against a heightened anxiety baseline. These findings also establish a robust platform for high‐throughput identification of anxiolytic compounds with potential relevance for treating anxiety and related stress in humans.

## Graphical abstract

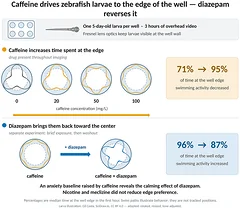

## 1 Introduction

In humans, anxiety disorders are characterized by their tendency to interfere with daily activities such as work, school, and personal relationships. Generalized anxiety disorder, agoraphobia, social anxiety disorder, and post‐traumatic stress disorder (PTSD) are a few of the many anxiety disorders that plague the United States, with recent statistics from the National Institute of Mental Health showing that 19.1% of adults had an anxiety disorder in the past year and 31.1% of adults have experienced anxiety disorders at some point in their lives. The statistics are similar for adolescents (13–18 years old), with an estimated 31.9% of adolescents having an anxiety disorder (National Institute of Mental Health (NIMH) n.d.). A study on the relationship between caffeine and anxiety showed that high doses of caffeine, especially above 400 mg, caused a highly significant increase in the risk of anxiety in individuals not previously diagnosed with any anxiety disorders (Liu et al. 2024). Considering that one specialty coffee can contain more than 475 mg of caffeine (Naidoo 2020), and 85% of the US population consumes at least one caffeinated beverage per day (Mitchell et al. 2014), caffeine overconsumption is a significant problem in the United States. Despite this, no validated behavioral platform exists for screening compounds against caffeine‐induced anxiety specifically, leaving a gap between the scale of the clinical issue and the available tools to address it.

Zebrafish are commonly used as an animal model for testing behavioral and neurochemical effects of pharmaceuticals due to their ability to mirror drug‐induced behaviors and complex human brain disorders such as Alzheimer's (Kalueff et al. 2014). The human adenosine system contains four receptor subtypes: A1, A2A, A2B, and A3. When normal caffeine doses are consumed, caffeine binds to A1 and A2A receptors with high affinity, acting as an antagonist and preventing adenosine binding (Fredholm et al. 2005). In addition to affecting sleep, arousal, and memory, A1 and A2A receptors modulate other neurotransmitters like GABA, serotonin, and dopamine, which can cause anxiety‐inducing effects (Maximino et al. 2011). Just like humans, zebrafish possess the adenosine receptor subtypes A1, A2A1, A2A2, and A2B, along with the phosphoprotein DARPP‐32 (Capiotti et al. 2011). Caffeine has not been seen to cause any morphological alterations in zebrafish at concentrations used in prior behavioral studies; instead, it only affects the previously discussed cellular targets (Capiotti et al. 2011). These features make zebrafish a suitable model for determining behavioral changes induced by caffeine.

Extensive prior research has shown that caffeine exposure in zebrafish causes increased anxiety‐like behaviors, such as increased thigmotaxis, across a broad range of doses and behavioral endpoints (Rosa et al. 2018; Li et al. 2024; Richendrfer et al. 2012; Clayman and Connaughton 2022). While the side‐view Novel Tank Diving Test is a standard assay for quantifying vertical anxiety‐like behaviors in zebrafish, the method limits scalability and high‐throughput parallel screening, causing researchers to pivot to overhead‐camera configurations with multi‐well plates (Rihel et al. 2010; Kokel et al. 2010; Gore et al. 2023; Scholz et al. 2025). A persistent challenge with this solution lies in the optical artifacts present in the behavioral arenas, with severe edge distortion creating optical blind spots that lead to errors in even the most sophisticated machine‐learning tracking algorithms. Together, inconsistent thigmotaxis measurements, poor scalability in side‐view assays, optical artifacts in multi‐well tracking, and contradicting reports of diazepam's efficacy display that no current platform can achieve high scalability while also producing reproducible, artifact‐free anxiety measurements. To address these limitations, our study utilizes a Fresnel lens and AI‐assisted tracking through a modified version of the Zebrafish Larvae Position Tracker (Z‐LaP Tracker) (Gore et al. 2023) in larger six‐well plates. Furthermore, since prior work has found conflicting results when assessing diazepam and other benzodiazepines’ anxiolytic ability in a zebrafish model (Zahid et al. 2018; Bencan et al. 2009), we leverage this artifact‐free tracking array to isolate diazepam's GABAergic rescue of caffeine‐induced thigmotaxis and validate the assay using a known anxiolytic. By tracking a variety of behaviors, including overall activity, sound startle, optomotor response, scoot, burst, orientation, and turn, in 5‐day‐old zebrafish across a wide range of caffeine doses (0–100 mg/L), we validate a highly scalable, high‐fidelity platform for screening novel anxiolytics. We further show that an elevated‐anxiety baseline reconciles long‐standing inconsistencies in diazepam's reported efficacy in zebrafish.

## 2 Materials and Methods

### 2.1 Experimental Animals

Zebrafish husbandry and maintenance were performed as described in a prior study by our lab (Gore et al. 2023), with minor modifications. All experiments carried out in this study followed federal regulations and guidelines for the ethical and humane use of animals and have been approved by Brown University's Institutional Animal Care and Use Committee (IACUC). All experiments were carried out using 5‐day postfertilization (dpf) zebrafish larvae. Adult zebrafish (*Danio rerio*) are maintained in the Animal Care Facility at Brown University as a genetically diverse wild type strain with a mixed population of males and females. The zebrafish are maintained on a 14‐h light, 10‐h dark cycle in a Marineland Vertical Aquatic Holding System. The zebrafish adults are fed daily with Gemma Micro 300 and frozen brine shrimp at noon during the light cycle. Embryos were collected at “dawn” and raised in 2‐L breeder tanks at 28.5°C in egg water (60 mg/L instant ocean sea salt and 0.25 mg/L methylene blue in deionized water). The embryos hatched from their chorion at 3 dpf and started free swimming from 3–4 dpf. At 5 dpf, larvae do not need external food sources as they consume nutrients available in the yolk sac (Clift et al. 2014).

### 2.2 Experimental Design

Treatment groups and controls were imaged on the same day in the same imaging cabinet, with repeat experiments conducted on different days. A sample size of 96 was initially used for the caffeine‐alone groups, but a sample size of 48 was found to be sufficient and was used for diazepam–caffeine treatment groups. The sample size was informed by the effect size in the caffeine‐alone experiment (rank‐biserial *r* = 0.344, control vs. 100 mg/L caffeine on edge preference). A power analysis indicated that *n* = 43 was required to achieve 80% power at *α* = 0.05 (two‐sided Mann–Whitney *U* test). A sample size of *n* = 48 was selected to provide 84.7% power. For each of the treatment groups, larvae were obtained from at least three independent clutches after group breeding and randomly assigned to the treatment groups. In each caffeine imaging experiment, an egg water control group was included. In prior studies, circadian rhythms have been seen to affect behavioral analysis (Barnard and Nolan 2008), so imaging was consistently performed in the 2–6 p.m. time window.

### 2.3 Pharmacological Treatments

Three exposure timelines were used to fully characterize caffeine‐induced behavioral changes (Figure 1). A sustained exposure protocol was employed to establish caffeine anxiogenic stability, observe behavioral stability across time, and monitor for exhaustion across a range of concentrations. For the diazepam rescue assays, an acute exposure protocol was used to maximize assay sensitivity (Schnörr et al. 2012). The caffeine and nicotine co‐treatment experiments utilized a pulse‐plus‐washout protocol to limit nicotinic acetylcholine receptor desensitization, further described in the Supporting Information. These differing drug exposure timelines were chosen to optimize anxiogenic ability based on established literature, rather than using a single, standardized protocol.

**FIGURE 1:**
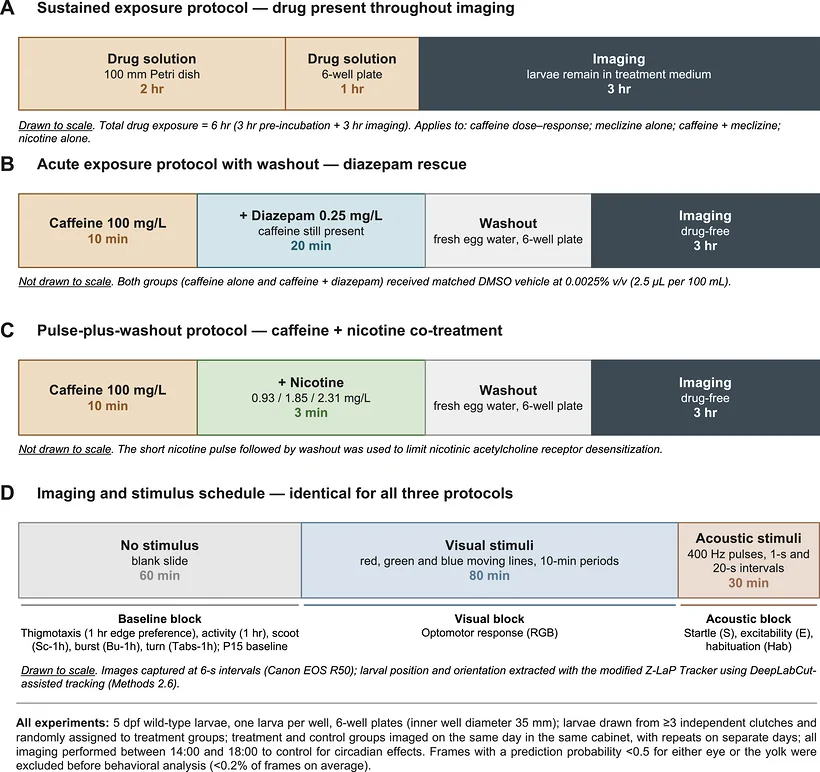
Experimental design. (A) Sustained exposure protocol, drawn to scale: larvae were preincubated in treatment solution for 2 h in Petri dishes and 1 h in six‐well plates, then imaged for 3 h with treatment medium present throughout. (B) Acute exposure protocol with washout, used for diazepam rescue. (C) Pulse‐plus‐washout protocol, used for caffeine + nicotine co‐treatment. Panels B and C are not drawn to scale. (D) Imaging and stimulus schedule, drawn to scale and identical across all three protocols, showing the behavioral measures derived from each block.

In the sustained exposure timeline, 5 dpf larvae were incubated in caffeine treatment solutions for 2 h in 100‐mm clear lid Petri dishes (Fisher Scientific, FB0875712) before being transferred to six‐well clear nontreated culture plates (Falcon, 351146) and incubated for another hour in the same treatment medium. After the 3‐h incubation period was completed, the six‐well plates were placed in the imaging cabinet. Although our lab's drug library screening was conducted in 96‐well plates ( Del Rosario Hernandez et al. 2024), six‐well plates were selected due to their ability to reduce confounding between edge‐hugging behavior and random movement. In the acute exposure protocol, 5 dpf larvae were incubated in 100 mg/L caffeine treatment solution for 10 min before adding 0.25 mg/L diazepam (CAS 439‐14‐5, Sigma D0899‐100 mg diazepam). After an additional 20‐min incubation period, the fish were transferred to six‐well plates in fresh egg water and immediately placed in the imaging cabinet.

For the caffeine‐only experiments, caffeine (CAS 58‐08‐2, Sigma C0750‐100 g) was dissolved directly in egg water to prepare 20 mg/L, 50 mg/L, and 100 mg/L solutions. Egg water without caffeine was used as a control. In the caffeine‐diazepam co‐treatment rescue experiments, the treatments were 100 mg/L caffeine alone and 100 mg/L caffeine with 0.25 mg/L diazepam prepared with stocks dissolved in DMSO. Both groups received 2.5 µL DMSO per 100 mL final volume (0.0025% v/v) as a vehicle control. A concentration of 0.25 mg/L diazepam was selected using a dose‐finding experiment (Section 3.2) in which 0.05, 0.1, 0.25, and 0.5 mg/L were compared against DMSO‐treated controls for effects on locomotor activity. Note that 0.25 mg/L was the highest concentration that did not significantly reduce activity relative to controls (*W* = 127, *p* = 0.58, *r* = 0.118), whereas 0.5 mg/L caused pronounced sedation (*W* = 36, *p* < 0.001, *r* = 0.750). This range is consistent with results from previous studies (Schnörr et al. 2012).

### 2.4 Imaging Setup

The imaging system for automated behavioral analysis contains four six‐well plates for 24‐well behavioral profiling. The setup consists of a high‐resolution camera (Canon EOS R50 with RF‐S18‐45 mm F4.5‐6.3 IS STM lens), white stage, projector (M5 LED pico projector, Aaxa Technologies, 900 lumens LED light source), Fresnel lens (300 × 300 mm lens, 600 mm focal length, Amazon B0D5BSQVTM), speakers (OfficeTec USB Computer Speakers Compact 2.0 System set to 85 dBA), and a laptop computer (Figure 2). A similar imaging system was used as in a previous study by our lab (Gore et al. 2023), with a few changes to optimize the setup for six‐well plates. The six‐well plates had clear bottoms, so we had to use a translucent white stage for the projection of the visual stimuli. Additionally, the angled view of the outer wells resulted in shadows and “blind spots” where the edge‐hugging zebrafish could not be seen. The addition of a Fresnel lens to the imaging setup resolved this issue (Figure 3). The projector was displaying a custom‐made Microsoft PowerPoint presentation used when previously screening a library of compounds (Del Rosario Hernandez et al. 2024). The camera settings used for the current study were ISO200, Fluorescent, F5, and 1/5 exposure.

**FIGURE 2:**
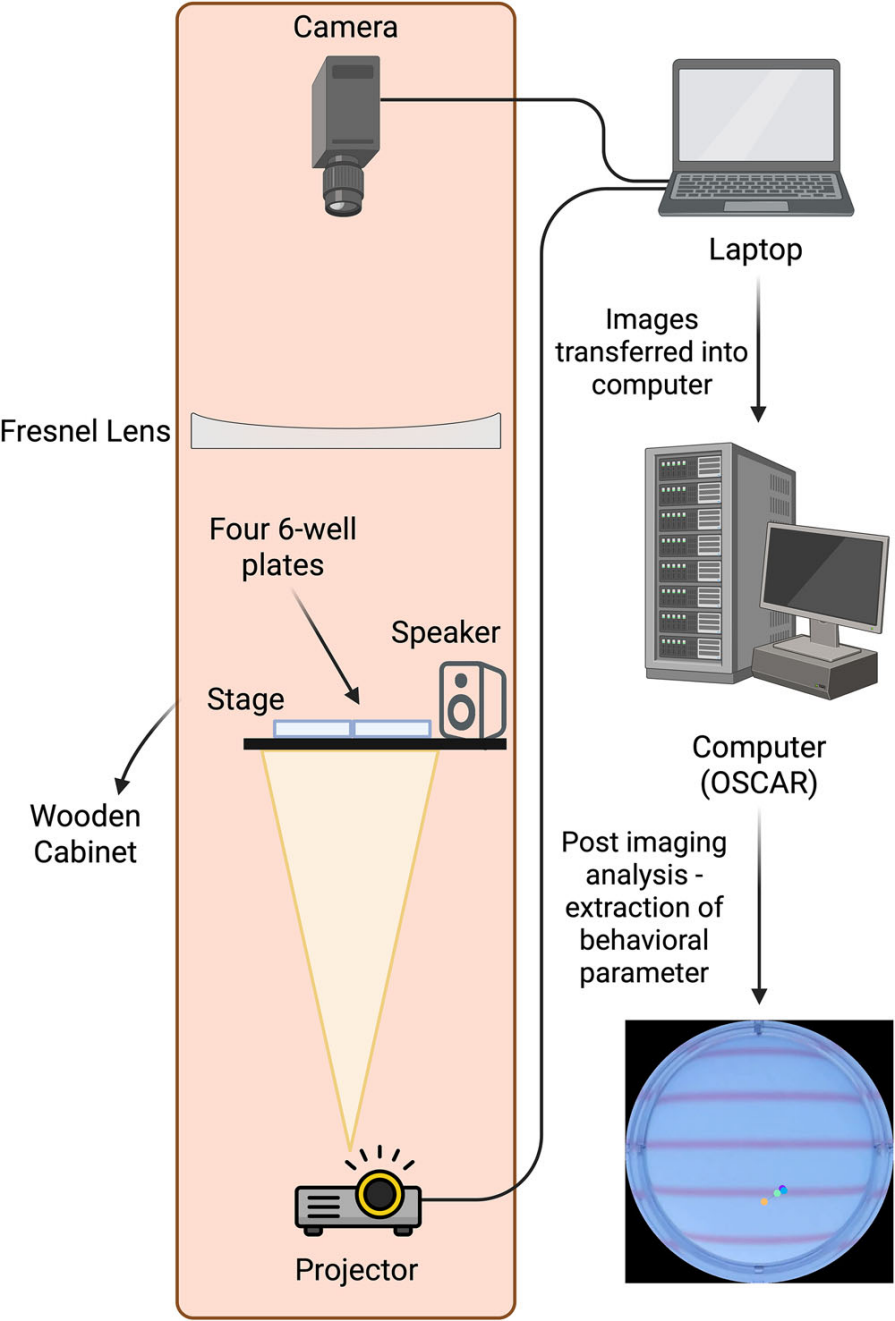
Experimental setup. The imaging setup consists of a projector attached to a laptop using PowerPoint for presenting visual/acoustic stimuli, a staging area for four six‐well plates, and a high‐resolution camera that takes photos of the multi‐well plates at 6‐s intervals. The Fresnel lens is included to reduce blind spots and shadows along the edges of the wells. The data are analyzed post‐imaging using an automated python pipeline running on Brown University's OSCAR computing system.

**FIGURE 3:**
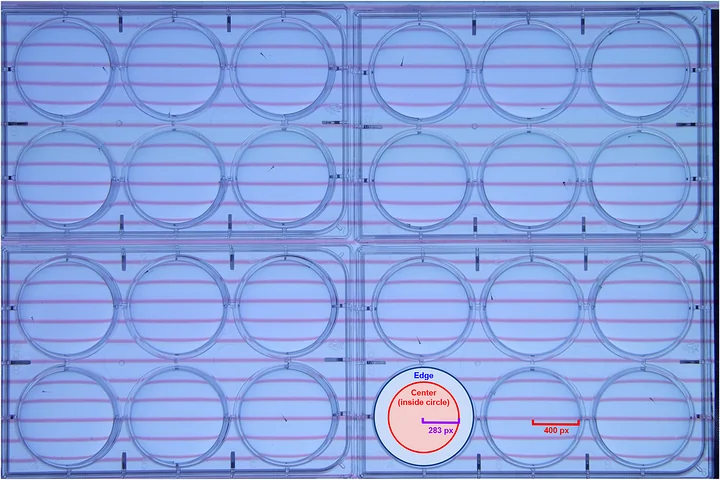
Top–down image of six‐well plates as taken by the camera in the imaging cabinet and processed by OSCAR. Moving red lines are presented as visual stimuli. Zebrafish larvae move in the same direction as the visual stimuli (optomotor response). Each well contains one zebrafish larva. Inner diameter of the wells = 35 mm.

### 2.5 Behavioral Assay

The behavioral assay was identical to the previous study when screening a library of drug compounds (Del Rosario Hernandez et al. 2024). To summarize, a 3‐h long PowerPoint presentation consisted of 1 h of no stimulation (blank slide) followed by 80 min of visual stimuli in the form of red, green, and blue lines moving up and down in 10‐min periods. The presentation concluded with 30 min of acoustic stimuli in the form of 400 Hz sound pulses with 1‐ or 20‐s intervals. Using the same PowerPoint presentation allowed us to compare our results to the drug library screening and select compounds to test, such as meclizine and nicotine. Meclizine, an H1 receptor antagonist, was selected to test whether histaminergic modulation alters caffeine‐induced thigmotaxis. Nicotine, a nicotinic acetylcholine receptor agonist with a reported biphasic anxiolytic‐to‐anxiogenic dose response, was selected to test whether a non‐GABAergic stimulant could rescue caffeine‐induced thigmotaxis. Results for both compounds are reported in the Supporting Information and summarized in Section 3.3.

### 2.6 Automated Image Analysis

To analyze six‐well plates, we modified our lab's previous image processing framework Z‐LaP Tracker (Gore et al. 2023). Unlike Z‐LaP tracker, which was only able to analyze 96‐well plates, the modified version can analyze any multi‐well plate configuration, create labeled videos using DeepLabCut AI‐assisted tracking to analyze point labeling accuracy, and perform background masking outside wells to reduce labeling error (Figure 4). All details about the information, usage, and installation of the modified Z‐LaP tracker as well as an instructional video can be found at the GitHub repository (https://github.com/realtofuine/Manual‐Selection‐Analysis) and is permanently archived on Zenodo, v1.0.0 (Rai 2026). We used Brown University's access to the high‐performance computing cluster OSCAR (Ocean State Center for Advanced Resources) for analyzing zebrafish behavior, which allowed the analysis of one complete experiment to be completed in around 40 min. The output files as well as data processing and behavioral analysis remain identical to the original Z‐LaP tracker (Gore et al. 2023). Any images with a prediction probability of < 0.5 for any of left eye, right eye, or yolk are not used for behavioral calculations as *p* < 0.5 is considered an unreliable identification of larval features by the image processor. *p* < 0.5 was observed for an average of less than 0.2% of images. Thigmotaxis was calculated as the percentage of time that the yolk of a larva was located more than 12.37 mm (283 pixels) from the center of the well, or in the “edge” region of the well, during the 1st h of imaging. This value was determined by making the “center” region and “edge” region have equal areas (around 481 mm^2^ each) as shown in Figure 3. The equal area boundary was selected a priori as a null hypothesis threshold, with a fish being expected to spend equal time (50%) in each zone by chance. This avoids a threshold‐tuned post hoc to a particular dataset or effect size. Activity (1 h) was calculated as the percentage of time that a larva moves more than three pixels between consecutive images within the 1st h before being exposed to any stimuli. Burst (Bu‐1 h) was calculated by determining the percent of time that the fish moved more than 20 pixels between consecutive images, while scoot (Sc‐1 h) was the percent of time that the fish moved between 3 and 20 pixels between consecutive images. Excitability (E) was determined as the percent change in activity during the 1‐s sound interval period compared to the 20‐s sound interval period. Negative excitability values indicate that activity was lower during the 1‐s interval period than during the 20‐s interval period. Startle (S) was the percent change in activity during the 20‐s sound interval period compared to baseline activity (P15), where P15 denotes the average activity during the no‐sound period. Habituation (Hab) was the percent change in activity during the first 5 min of the 1‐s sound period to the second 5 min, with higher values indicating higher habituation. The turning behavior (Tabs‐1 h) was determined by calculating the absolute value of the difference in angle of the fish (using eyes and yolk position) and averaging the values. The vision values (RGB) were the combined optomotor response with moving lines of any color or speed, calculated by determining the average difference in time spent in the upper part of the well between downward‐moving line periods and upward‐moving line periods. These additional behaviors were measured because previous studies have discovered that anxiety‐related behaviors are modified by visual and auditory stimuli (Richendrfer et al. 2012; Burgess and Granato 2007; Colwill and Creton 2011).

**FIGURE 4:**
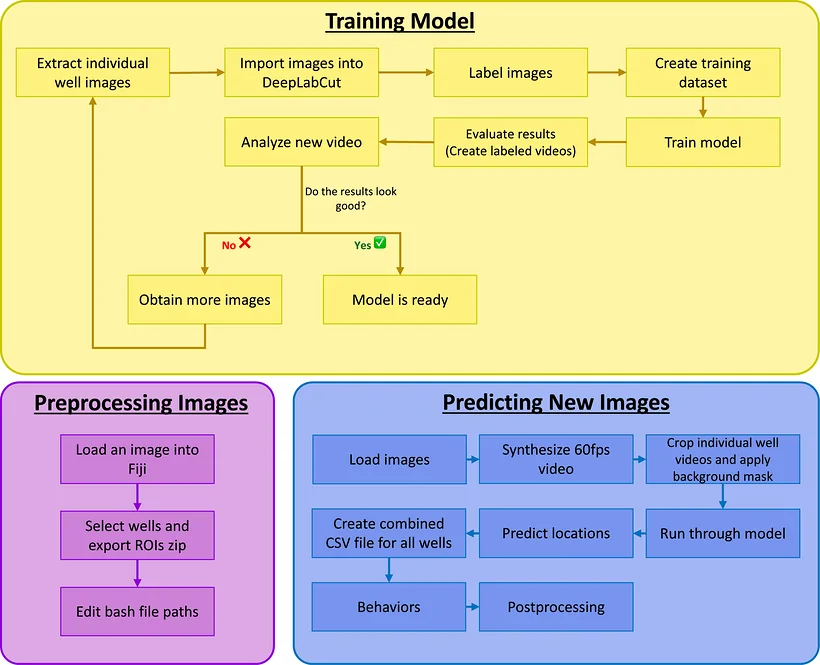
Flowchart for automated behavioral analysis. The model was trained on a set of images and used to predict new images by isolating each well, applying a filter background mask, and running them through the image analysis model.

### 2.7 Statistical Analysis

All of the statistical tests and data visualization were conducted using Microsoft Excel (version 16.110), custom Python (version 3.14) scripts, and R (version 4.5.1). R was used for statistical analyses and graphs, Python was used in the image analysis pipeline, and Excel was used for data tabulation. Images with a prediction probability < 0.5 for either eye or yolk were excluded before behavioral analysis (affecting < 0.2% of images on average). Median values with first and third quartiles (Q1–Q3) are shown in the graphs and text. Since the values of our data set were not normally distributed as seen in *Q*–*Q* plots, had unequal sample sizes per experimental group, and had unequal between‐group variance, we used nonparametric Kruskal–Wallis one‐way ANOVA with post‐hoc Dunn's test only following a significant Kruskal–Wallis result and the two‐sided Mann–Whitney *U* test. Effect sizes were calculated for each comparison, *ε* ^2^ for Kruskal–Wallis tests and rank‐biserial correlation (*r*) for Mann–Whitney *U* and Dunn's pairwise tests. A Bonferroni correction was applied for multiple comparisons, with *α* = 0.0125 for the four group diazepam dose‐finding comparison and post‐hoc Dunn's tests conducted using the Bonferroni method. Each behavior was treated as a mechanistically distinct outcome. Power analysis for sample size determination was conducted using the shiehpow function in the wmwpow package (R, version 0.1.3). All statistical tests were two‐sided, with significance set a priori at *α* = 0.05 unless otherwise noted. Differences in behaviors were considered significant when *p* < 0.05.

## 3 Results

Complete results files with data for all measured behaviors are available in the Supporting Information.

### 3.1 Caffeine

Exposure to caffeine alone caused an increase in the percentage of time spent near the edge, with higher caffeine concentrations corresponding to higher edge preference (*H*(3) = 52.08, *p* < 0.001, *ε* ^2^ = 0.139) (Figure 5). The control group (0 mg/L caffeine) had the lowest edge preference (71.45% [52.38–87.12], *n* = 94), followed by 20 mg/L caffeine (79.03% [54.63–92.73], *n* = 95), 50 mg/L caffeine (86.83% [71.68–97.73], *n* = 94), and finally 100 mg/L caffeine (94.51% [87.28–98.62], *n* = 94). The difference in edge preference between the control and 100 mg/L caffeine‐treated groups is significant (Dunn's test *p* < 0.001). When comparing the control group versus the 100 mg/L caffeine group, the caffeine group displayed statistically significant lower activity overall (caffeine: 66.59% [35.85–88.67] vs. control: 87.68% [58.10–97.53]; *H*(3) = 24.39, *p* < 0.001, *ε* ^2^ = 0.065; Dunn's test *p* < 0.001), lower optomotor response (13.95% [2.05–24.02] vs. 18.90% [10.63–36.08]; *H*(3) = 26.92, *p* < 0.001, *ε* ^2^ = 0.070; Dunn's test *p* = 0.003; see Discussion for activity‐controlled analysis), higher excitability (1.67% [−3.72 to 13.66] vs. −3.10% [−26.00 to 2.00]; *H*(3) = 21.88, *p* < 0.001, *ε* ^2^ = 0.057; Dunn's test *p* < 0.001), higher scoot (25.71% [15.51–38.85] vs. 10.61% [2.77–19.29]; *H*(3) = 52.44, *p* < 0.001, *ε* ^2^ = 0.139; Dunn's test *p* < 0.001), lower burst (27.51% [12.03–49.07] vs. 70.04% [35.39–93.32]; *H*(3) = 50.06, *p* < 0.001, *ε^2^* = 0.133; Dunn's test *p* < 0.001), and lower turning behavior (25.71° [16.48–38.22] vs. 43.23° [27.15–60.37]; *H*(3) = 31.12, *p* < 0.001, *ε* ^2^ = 0.083; Dunn's test *p* < 0.001). To view location and activity simultaneously, swim patterns were displayed in ‘X, Y, Time’ graphs (Figure 6). Overall, these experiments show that 100 mg/L caffeine induces various changes in behavior, including suppressed activity and elevated thigmotaxis.

**FIGURE 5:**
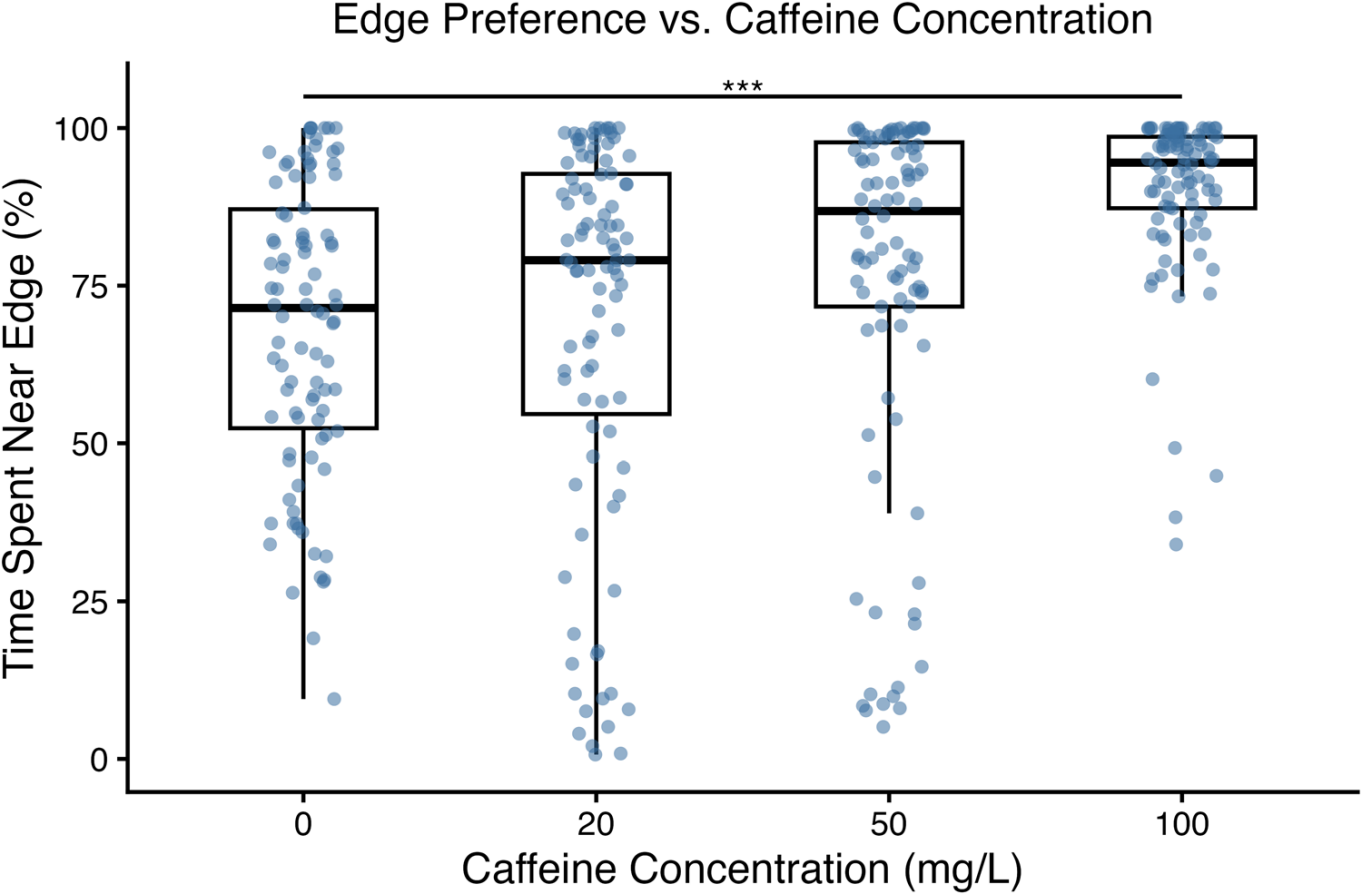
Percent of time spent near the edge of the well during the 1st h of recording compared across control 0‐ (*n* = 94), 20‐ (*n* = 95), 50‐ (*n* = 94), and 100‐mg/L (*n* = 94) caffeine treatment groups. Boxes show median and interquartile range, with individual data points overlaid. Edge preference increased as caffeine concentration increased (Kruskal–Wallis *H*(3) = 52.08, *p* < 0.001, *ε* ^2^ = 0.139; 100 mg/L vs. control, Dunn's post‐hoc *p* < 0.001). ***p < 0.001.

**FIGURE 6:**
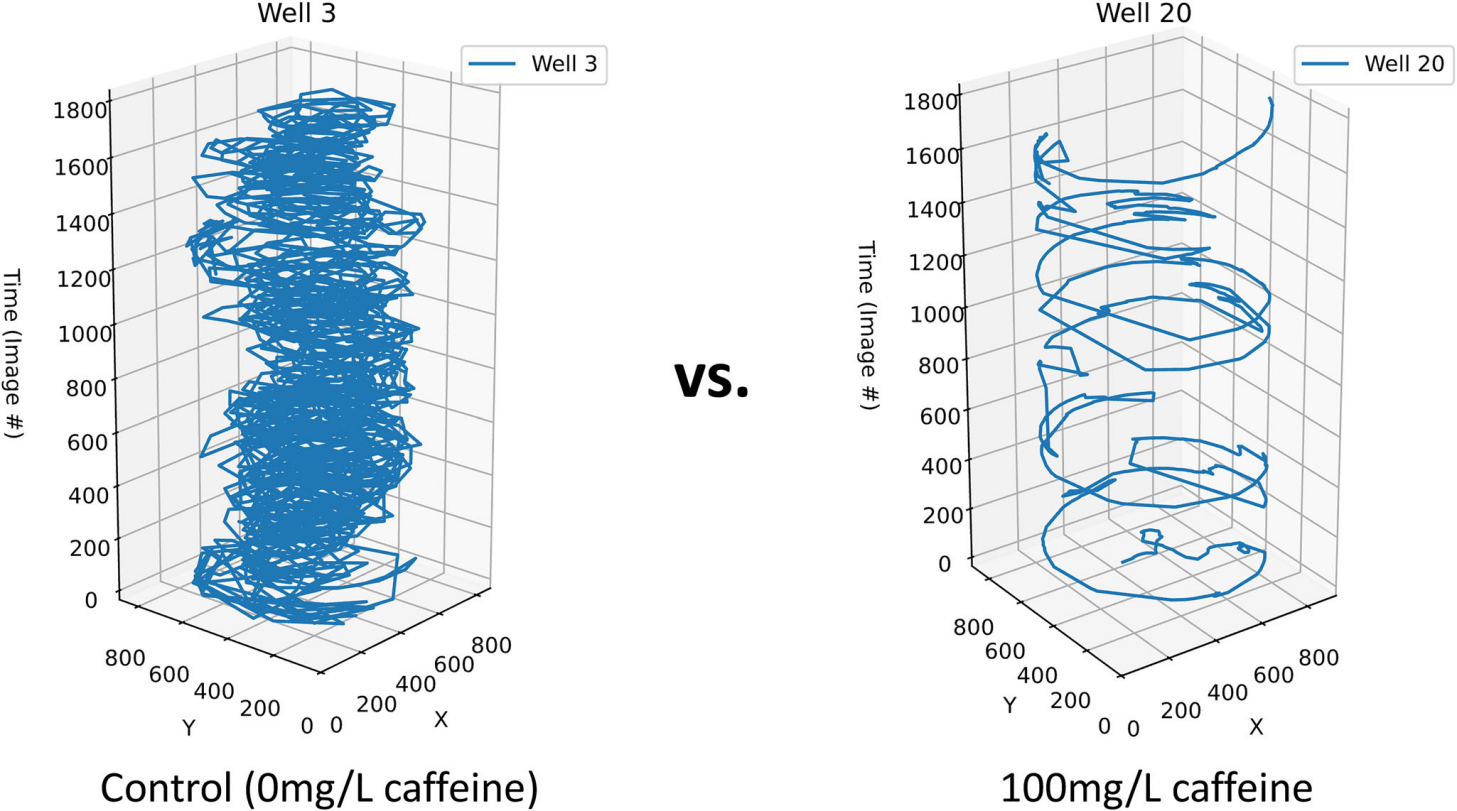
Zebrafish behavior displayed in “X, Y, Time” graphs. X and Y position in the well as a function of time (Z axis). Examples of an untreated control and a 100‐mg/L caffeine‐treated zebrafish larva. The caffeine‐treated zebrafish larva displays more edge‐hugging behavior and lower activity than the control fish.

### 3.2 Diazepam

In an initial dose‐finding experiment, we treated zebrafish larvae with 0.05, 0.1, 0.25, and 0.5 mg/L diazepam (*n* = 24 for DMSO pooled across two sessions, *n* = 12 for the other groups). Activity had a median of 49.76% (30.42–59.65) in the DMSO‐treated controls versus 43.17% (37.44–55.92) (*W* = 124, *p* = 0.51, *r* = 0.139), 38.36% (35.99–44.57) (*W* = 102, *p* = 0.30, *r* = 0.292), 43.30% (35.07–52.04) (*W* = 127, *p* = 0.58, *r* = 0.118), 13.20% (11.25–19.35) (*W* = 36, *p* < 0.001, *r* = 0.750), in the 0.05‐, 0.1‐, 0.25‐, and 0.5‐mg/L diazepam treatment groups, respectively (Bonferroni‐corrected *α* = 0.0125 for four comparisons). We selected 0.25 mg/L for subsequent rescue experiments, as this was the highest dose that did not decrease activity compared to controls.

Zebrafish larvae were treated with caffeine or caffeine + diazepam (*n* = 48 larvae per group). The rescue group with diazepam displayed a lower edge preference (Figure 7). Edge preference had a median of 95.95% (92.01–98.90) in the caffeine group versus 87.48% (77.68–94.61) in the caffeine + diazepam group (*W* = 547, *p* < 0.001, *r* = 0.525). In addition, the rescue group displayed higher activity overall (caffeine + diazepam: 57.85% [51.49–72.04] vs. caffeine: 45.26% [38.20–57.84]; *W* = 1700, *p* < 0.001, *r* = 0.476), higher activity in response to sound (68.46% [53.75–76.79] vs. 54.56% [38.75–70.00]; *W* = 1527, *p* < 0.001, *r* = 0.326), higher burst movements (29.04% [22.56–47.13] vs. 12.93% [9.33–19.19]; *W* = 1924, *p* < 0.001, *r* = 0.670), and higher turning behavior (25.96° [21.51–36.76] vs. 16.14° [11.82–21.71]; *W* = 1878, *p* < 0.001, *r* = 0.630). The increase in activity (Figure 8) indicates that the reduction in edge preference was not due to motor suppression. Overall, these experiments show that diazepam can rescue caffeine‐induced changes in activity and thigmotaxis.

**FIGURE 7:**
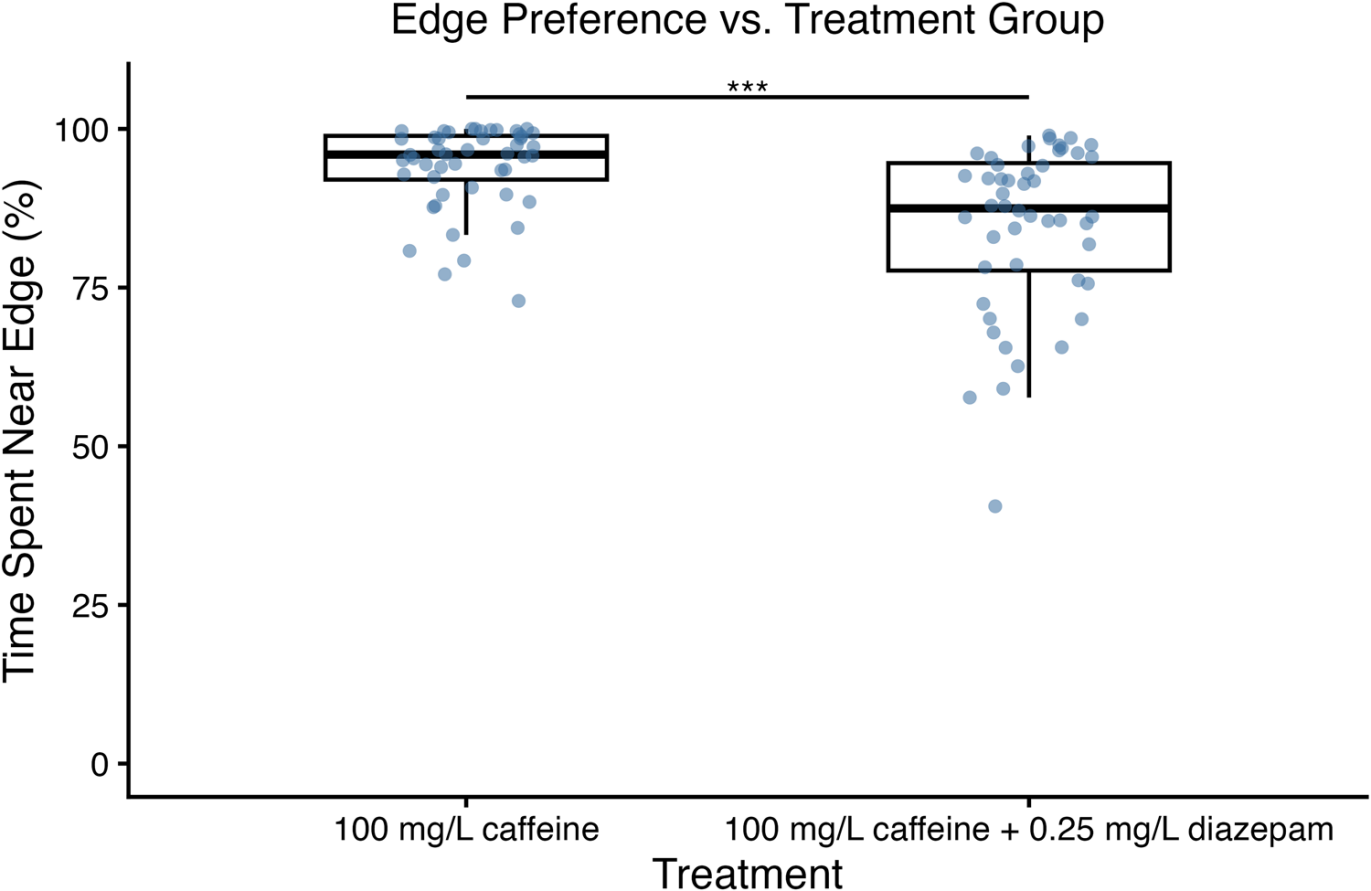
Percent of time spent near the edge of the well in the 1st h of recording compared between 100‐mg/L caffeine treatment group and rescue 100 mg/L caffeine with 0.25‐mg/L diazepam treatment group (*n* = 48 per group). Boxes show median and interquartile range, with individual data points overlaid. Edge preference for the rescue group was significantly lower than that for the 100‐mg/L caffeine group (Mann–Whitney *W* = 547, *p* < 0.001, rank‐biserial *r* = 0.525). ***p < 0.001.

**FIGURE 8:**
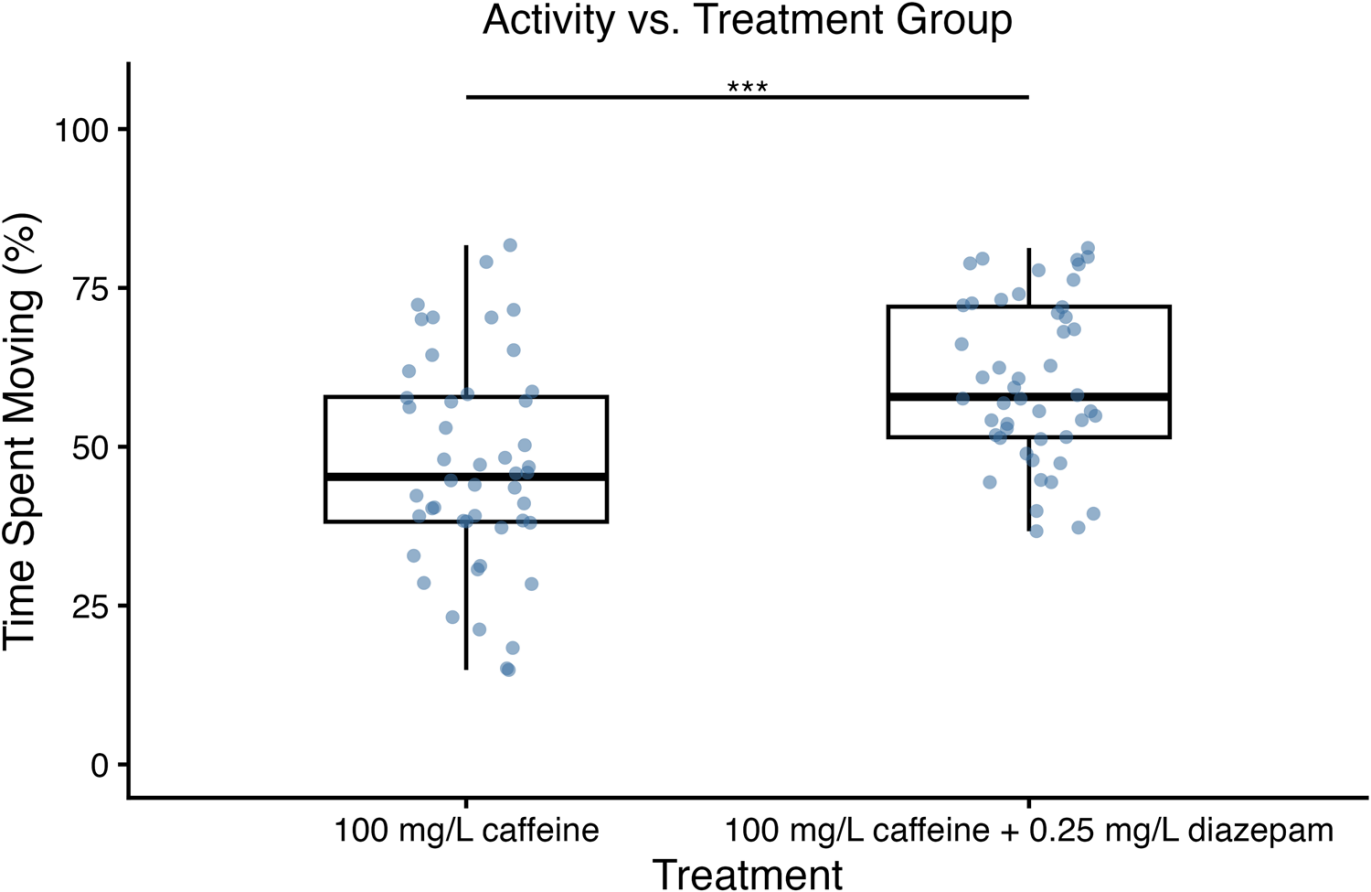
Activity (percent of time spent moving in the 1st h of recording) compared between 100‐mg/L caffeine treatment group and rescue 100 mg/L caffeine with 0.25‐mg/L diazepam treatment group (*n* = 48 per group). Boxes show median and interquartile range, with individual data points overlaid. Activity for the rescue group was significantly higher than that for the 100‐mg/L caffeine group (Mann–Whitney *W* = 1700, *p* < 0.001, rank‐biserial *r* = 0.476). ***p < 0.001.

### 3.3 Meclizine and Nicotine

Meclizine and nicotine were examined to test whether caffeine‐induced thigmotaxis could be rescued by non‐GABAergic mechanisms (Figure 9). Nicotine produced no significant change in edge preference alone (*H*(3) = 1.65, *p* = 0.64, *ε* ^2^ = 0.036) or in combination with 100‐mg/L caffeine (*H*(3) = 1.23, *p* = 0.75, *ε* ^2^ = 0.009), and did not alter overall activity (*H*(3) = 0.58, *p* = 0.90, *ε* ^2^ = 0.004). Meclizine alone increased edge preference at 0.5 mg/L (*H*(3) = 15.26, *p* = 0.002, *ε* ^2^ = 0.162; Dunn's *p* = 0.01), but this dose also suppressed activity, preventing attribution to an anxiogenic mechanism. In combination with caffeine, 0.25 mg/L meclizine increased edge preference during the visual lines period (*W* = 124, *p* = 0.01, *r* = 0.508) without altering 1st‐h thigmotaxis (*W* = 171, *p* = 0.11, *r* = 0.321). Neither compound reduced caffeine‐induced edge preference at any tested concentration. Full results are reported in the Supporting Information.

**FIGURE 9:**
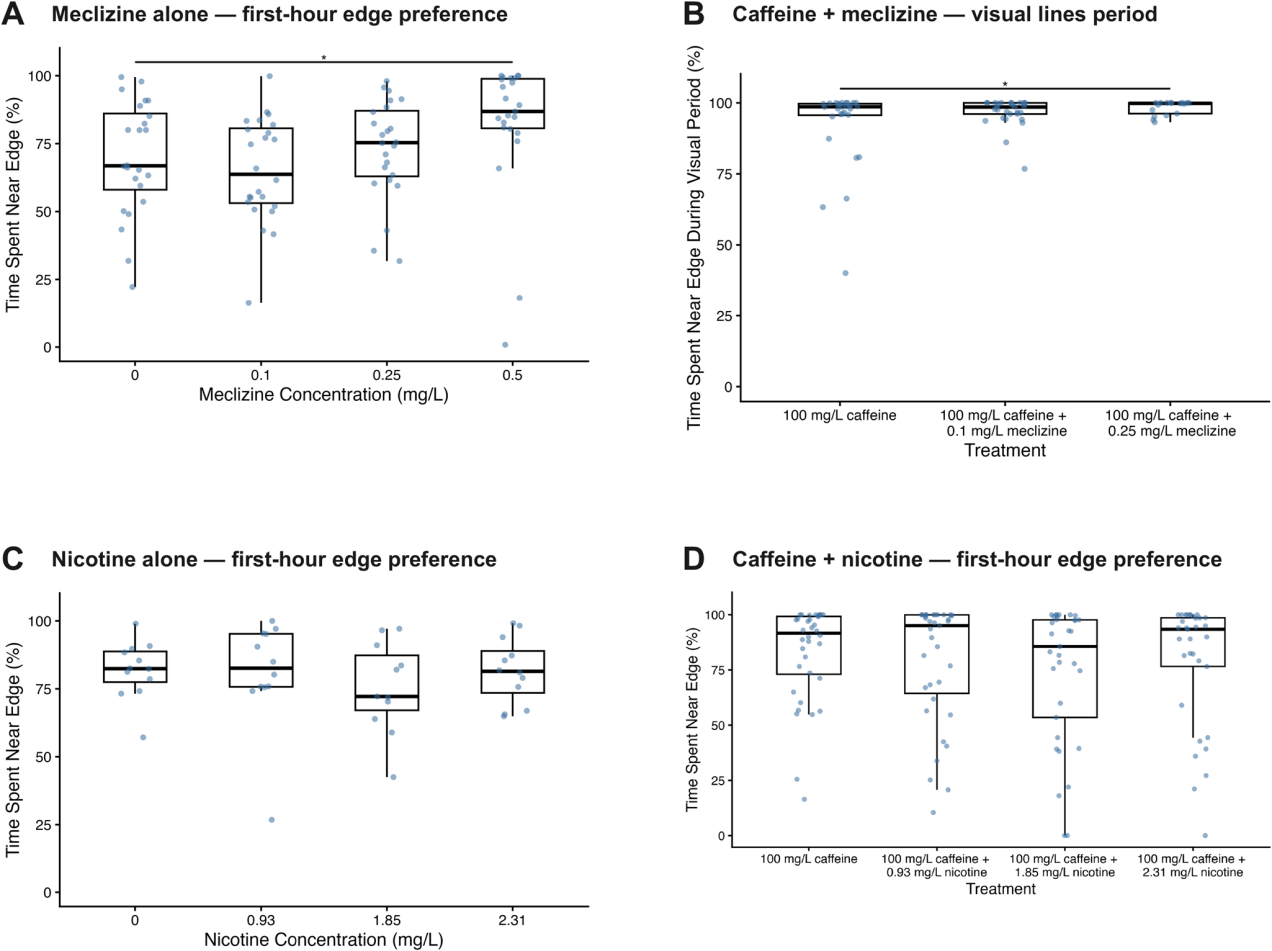
Effects of meclizine and nicotine on edge preference in 5 dpf zebrafish larvae. (A) Meclizine alone at 0, 0.1, 0.25, and 0.5 mg/L; 1st‐h edge preference increased at 0.5 mg/L (Kruskal–Wallis *H*(3) = 15.26, *p* = 0.002, *ε* ^2^ = 0.162; 0.5 mg/L vs. control, Dunn's post‐hoc *p* = 0.01), a dose that also suppressed locomotor activity. (B) Effects of 100 mg/L caffeine alone or with 0.1 or 0.25 mg/L meclizine; edge preference during the visual lines period was higher with 0.25 mg/L meclizine than with caffeine alone (Mann–Whitney *W* = 124, *p* = 0.01, rank‐biserial *r* = 0.508). (C) Nicotine alone at 0, 0.93, 1.85, and 2.31 mg/L; no significant differences in 1st‐h edge preference (*H*(3) = 1.65, *p* = 0.64, *ε* ^2^ = 0.036). (D) Effects of 100 mg/L caffeine alone or with 0.93, 1.85, or 2.31 mg/L nicotine; no significant differences (*H*(3) = 1.23, *p* = 0.75, *ε* ^2^ = 0.009). Boxes show median and interquartile range with individual data points overlaid. (A) *n* = 24 per group; (B) *n* = 28, 29 and 17; (C) *n* = 12 per group; (D) *n* = 36 per group. *p < 0.05.

## 4 Discussion

The current study used a modified imaging system for the automated analysis of zebrafish larval behavior in four six‐well plates. We included a Fresnel lens so that larvae are always visible, even when they are close to the well walls. In addition, we adjusted the Z‐LaP Tracker's well extraction algorithm for AI‐assisted analysis of larval locations and orientations in six‐well plates. We used this modified imaging system to examine the effects of caffeine on a broad range of behaviors, including thigmotaxis and activity. We found that 100 mg/L caffeine increases thigmotaxis and reduces activity. The reduction in activity was accompanied by a shift in movement type, with increased scoot (low‐velocity movements), decreased burst (high‐velocity movements), and decreased turning. These behaviors are consistent with either a general sedative effect or a freezing‐like response, defined as episodes of immobility (Egan et al. 2009), with the increased scoot suggesting nonuniform immobility. Our measurements summarize movement over time bins rather than calculating specific immobility durations, so we cannot fully elucidate the mechanism. Additionally, caffeine‐treated fish had a reduced optomotor response, raising the possibility that optomotor response reflects suppressed locomotion rather than a change in visual processing. Activity and optomotor response have been previously shown to have separate effects in a similar behavioral profiling framework, with some compounds increasing activity while decreasing optomotor response and others decreasing activity while increasing optomotor response (Tucker Edmister et al. 2022). To determine if this held true for caffeine, a post hoc analysis restricting the comparison to caffeine‐treated larvae with comparable activity levels to controls (within control activity IQR) was conducted. The difference in optomotor response was no longer statistically significant (*W* = 1998, *p* = 0.23, *r* = 0.124), indicating that caffeine's reduction in optomotor response is attributable to suppressed locomotion. A similar comparison accounting for activity levels was conducted on edge preference, with the results indicating that the increased edge preference was not attributable to suppressed locomotion (*W* = 3550, *p* < 0.001, *r* = 0.574). It should be noted that these matched analyses had reduced power (*n* = 48 vs. 94).

Our behavioral observations of caffeine's effects align with the established biphasic pharmacological profile of caffeine in zebrafish models. Current literature demonstrates that while low‐to‐moderate doses of caffeine act as locomotor stimulants, exposure to high concentrations of caffeine (50–100 mg/L) causes anxiogenic behaviors (Cachat et al. 2010; Chen et al. 2021). Our Fresnel lens tracking system was able to capture this high‐dose transition to reduced activity and increased edge preference without confounding data loss at the well periphery, allowing us to establish a stable baseline level of anxiety to evaluate anxiolytic interventions.

Administering diazepam after 10 min of caffeine exposure reduced thigmotaxis significantly while also increasing overall activity levels, suggesting that diazepam rescues caffeine‐induced effects on behavior. Although the rescue experiment lacked an egg water control under the acute exposure protocol, the caffeine‐alone group reached a median edge preference of 95.95% compared to 94.51% in the sustained exposure protocol, consistent with anxiogenesis. Diazepam's efficacy in reducing thigmotaxis relies entirely on the within‐experiment comparison and does not require cross‐protocol analysis. Additionally, while the diazepam rescue dose was chosen from a small sample size dose‐finding experiment (*n* = 12), a significant sedative effect was still observed and dose selection was informed by previous work showing that diazepam is anxiolytic at doses as low as 0.05 mg/L, while doses greater than 5 mg/L produce pronounced sedation (Richendrfer et al. 2012). These reference concentrations come from differing exposure protocols and larvae ages but are presented as supporting context rather than direct validation of the dose selected.

As an adenosine antagonist, caffeine blocks adenosine receptors, causing an excitatory surge that results in anxiety and thigmotaxis (Richendrfer et al. 2012; Fredholm et al. 1999). On the other hand, diazepam is a positive allosteric modulator of the GABA‐A receptor, causing a higher chloride influx and therefore neuronal hyperpolarization, effectively raising the threshold for action potential initiation and restoring the central nervous system's excitation/inhibition balance (Campo‐Soria et al. 2006; Möhler 2012). Although the two drugs do not compete for the same receptors, they display physiological antagonism, resulting in decreased thigmotaxis, higher exploration, and higher overall activity (Tran et al. 2017).

Despite diazepam's status as a gold‐standard anxiolytic, its behavioral profile in zebrafish larvae has been inconsistent across existing literature. Although it had been previously found that diazepam has a significant reduction in edge preference in larval zebrafish (Richendrfer et al. 2012; Schnörr et al. 2012), recent reproducibility studies indicate that diazepam efficacy is highly sensitive to biological variables including sex (Genario et al. 2019) and strain‐specific baseline anxiety (Sackerman et al. 2010; Vignet et al. 2013). Studies have also had confounding between true anxiolysis and general locomotor suppression, with some finding that diazepam fails to reduce thigmotaxis at nonsedative doses (Zahid et al. 2018; Chen et al. 2021). These contradictions likely arise from the “floor effect” in low‐stress baselines. By using caffeine treatments to establish a high anxiety standard, our study demonstrates that diazepam functions as an anxiolytic agent. Additionally, previous studies not utilizing a Fresnel lens may underreport edge‐hugging behavior due to occlusion from the well plate itself.

The failure of both nicotine and meclizine to rescue caffeine‐induced edge preference, in contrast to diazepam, indicates that the thigmotaxis is not reversed by generalized stimulant or antihistaminergic action, therefore supporting a specific GABAergic mechanism of rescue. However, the nicotine experiments used *n* = 12 per group and are underpowered to detect small effects, so this comparison is interpreted as supportive rather than definitive.

This study has some limitations that warrant consideration. The diazepam rescue experiment lacked an acute‐protocol egg‐water control, with activity and edge preference in the caffeine + diazepam group being referenced against the caffeine‐alone group. This design isolates diazepam's effect relative to caffeine‐exposed high baseline anxiety but does not determine whether diazepam fully returns behavior to an unstressed baseline. Since the rescue experiment used the acute protocol, diazepam's effect should not be generalized to the sustained‐exposure baseline and limited to within‐experiment conclusions. The conclusions rest on the behavioral assay, and orthogonal anxiety‐related methodologies (novel tank diving test, light/dark preference) or physiological exams (cortisol levels) were not conducted and should be explored in future studies. The diazepam dose‐selection experiment was conducted with *n* = 12 per group, and although the large sedative effect at 0.5 mg/L was clearly and unambiguously detected, the precise sedating threshold requires further experimentation. The increase in edge preference under caffeine persisted after restricting comparison to activity‐matched subgroups, indicating that it is not solely due to suppressed locomotion. The thigmotaxis threshold was fixed a priori at the equal‐area boundary rather than tested with other radius cutoffs. While this avoids post hoc threshold selection, the sensitivity of edge preference to the selected boundary was not formally tested. These limitations do not affect the within‐experiment conclusions of the study showing that caffeine dose‐dependently increases edge preference while diazepam reverses this in a matched comparison, but they should limit how far the magnitude and mechanism of these effects are generalized.

In conclusion, this study validates the 5‐dpf zebrafish caffeine assay as a robust platform for detecting true anxiolysis over general sedation. By elevating baseline anxiety through caffeine administration, we reconciled conflicting reports regarding diazepam's efficacy, demonstrating that its anxiolytic properties are best seen against conditions of heightened arousal. This refined automated tracking system is a powerful tool to rapidly screen novel compounds for anxiolytic effects, potentially accelerating the discovery of accessible treatments for anxiety disorders, especially caffeine‐induced stress in humans.

## Author Contributions

**Rishi Rai**: conceptualization, methodology, software, formal analysis, investigation, writing – original draft. **Thais Del Rosario Hernandez**: procedure, resources, validation, writing – review and editing. **Robbert Creton**: supervision, funding acquisition, project administration, writing – review and editing.

## Funding

This work was supported by the NIH, NIGMS (R01GM136906). The supporting source had no role in study design; collection, analysis, and interpretation of data; writing of the report; or the decision to submit the report for publication.

## Conflicts of Interest

The authors declare no conflicts of interest.

## Supporting information




**Supplementary Material**: brb371769‐sup‐0001‐SuppMat.xlsx

**Supplementary Material**: brb371769‐sup‐0002‐SuppMat.xlsx

**Supplementary Material**: brb371769‐sup‐0003‐SuppMat.docx

## Data Availability

All data generated or analyzed during this study are included in this published article and its Supporting Information files.
