# Supplementary material for: Brewing Anxiety: Caffeine‐Induced Thigmotaxis in Zebrafish Larvae
Source: Brain Behav. 2026 Sep 30;16(10):e71769. doi: 10.1002/brb3.71769 (PMC13625816; doi:10.1002/brb3.71769)
Supplement: Supplementary file 1 [file BRB3-16-e71769-s002.docx]

Supplementary Data on Meclizine and Nicotine

# METHODS

## Experimental Design

The meclizine-only and nicotine-only experiments used the sustained-exposure timeline but with meclizine dihydrochloride (CAS 1104-22-9) and (-)-nicotine hydrogen tartrate (CAS 65-31-6). 0.1, 0.25, and 0.5 mg/L meclizine treatment groups and 0.93, 1.85, and 2.31 mg/L nicotine treatment groups were tested. Similarly, the meclizine and caffeine co-treatment experiments utilized the sustained-exposure timeline with concentrations of 100 mg/L caffeine combined with 0, 0.1, and 0.25 mg/L meclizine, chosen after preliminary toxicity experiments. However, the nicotine and caffeine co-treatment experiments were carried out using a pulse exposure with a washout protocol developed from previous nicotine studies in zebrafish that prevented receptor desensitization. After the zebrafish were exposed to 100 mg/L caffeine for 10 minutes, nicotine was added to concentrations of 0.93, 1.85, and 2.31 mg/L. 3 minutes later, the fish were transferred to clean egg water and immediately imaged for 3 hours.

***Statistical Methods***

All analyses followed Methods 2.7; caffeine+meclizine used two planned Mann-Whitney comparisons at Bonferroni-corrected α=.025.

# RESULTS

## 1. Meclizine

We first assessed the behavioral effects of meclizine dihydrochloride alone at 0.1, 0.25, and 0.5 mg/L. No significant differences were observed at 0.1 mg/L across any behavioral metrics (n = 24). At 0.25 mg/L, meclizine did not produce statistically significant changes either (n = 24). However, 0.5 mg/L meclizine significantly reduced several behaviors, including activity (meclizine: 59.38% [43.92–80.01] vs. control: 90.50% [86.71–91.35]; H(3) = 25.96, P<.001, ε^2^ = 0.276; Dunn’s test P<.001), burst (35.72% [20.78–48.47] vs. 87.57% [69.90–90.55]; H(3) = 31.61, P<.001, ε^2^ = 0.336; Dunn’s test P<.001), and turn (24.75° [15.85–33.58] vs. 57.64° [47.50–66.88]; H(3) = 32.75, P<.001, ε^2^ = 0.348; Dunn’s test P<.001), and was associated with increased scoot (22.27% [10.63–32.29] vs. 3.04% [0.68–8.49]; H(3) = 21.22, P<.001, ε^2^ = 0.226; Dunn’s test P<.001). Meclizine caused significant increase in thigmotaxis at 0.5 mg/L (86.83% [80.65–98.86] vs. 66.84% [58.03–86.09]; H(3) = 15.26, P=.002, ε^2^ = 0.162; Dunn’s test P=.01; see Discussion for confounding analysis) (Figure 1). These results indicated that low doses of meclizine are behaviorally inert, whereas higher doses affect thigmotaxis and cause other changes in behavior.

*Figure 1.* *Percent of time spent near edge of well compared between control 0mg/L, 0.1mg/L, 0.25mg/L, and 0.5mg/L meclizine treatment groups (n=24 for each). Boxes show median and interquartile range, with individual datapoints overlaid. A statistically significant difference in edge preference between 0mg/L meclizine and 0.5mg/L meclizine was observed (Kruskal-Wallis H(3) = 15.26, P=.002, ε^2^ = 0.162; 0.5mg/L vs. control, Dunn's post-hoc P=.01). * P<.05*

When combined with caffeine, there was no statistically significant difference in edge preference when comparing 100 mg/L caffeine vs. 100 mg/L caffeine + 0.1 mg/L meclizine (W = 377, P=.64, r = 0.071). There was a statistically significant increase in edge preference during the visual lines period when comparing 100mg/L caffeine vs. 100 mg/L caffeine + 0.25 mg/L meclizine (W = 124, P=.01, r = 0.508), but this effect was not present in first hour thigmotaxis (W = 171, P=.11, r = 0.321). First hour activity also did not differ significantly between the caffeine-only and caffeine + 0.25 mg/L meclizine groups (W = 302, P=.14, r = -0.198), indicating that the two groups did not differ in baseline motor state. Activity specific to the visual lines period was not available for this experiment, so we cannot exclude the possibility of sedation during that period contributing to the increase in edge preference. Given that this is a supplementary experiment, further experiments analyzing this effect would be recommended. We did not pursue 100 mg/L caffeine + 0.5 mg/L meclizine, as the larvae perished during extended observation (9 hrs), suggesting possible toxicity confounding during the 3 hour imaging period.

*Figure 2.* *Percent of time spent near edge of well during visual lines period compared between 100mg/L caffeine (n = 28), 100mg/L caffeine + 0.1mg/L meclizine (n=29), and 100mg/L caffeine + 0.25mg/L meclizine (n=17) treatment groups. Boxes show median and interquartile range, with individual datapoints overlaid. A statistically significant difference in edge preference between 100mg/L caffeine and 100mg/L caffeine + 0.25mg/L meclizine was observed (Mann-Whitney W=124, P=.01, rank-biserial r=0.508). * P<.05*

## 2. Nicotine

Nicotine was tested alone at concentrations of 0 mg/L (0µM), 0.93 mg/L (2µM), 1.85 mg/L (4µM), and 2.31 mg/L (5µM). The differences in edge preference were not statistically significant for any of the treatment concentrations (H(3) = 1.65, P=.64, ε^2^ = 0.036). 0 mg/L, 0.93 mg/L, 1.85 mg/L, and 2.31 mg/L nicotine groups (n = 12 for each) had median edge preferences of 82.42% [77.47–88.75], 82.6% [75.73–95.24], 72.18% [67.09–87.32], and 81.44% [73.49–88.93] respectively. Nicotine did not cause a statistically significant difference in any other behaviors.

*Figure 3.* *Percent of time spent near the edge of the well compared across control (0mg/L), 0.93mg/L, 1.85mg/L, and 2.31mg/L nicotine treatment groups (n=12 for each). Boxes show median and interquartile range, with individual datapoints overlaid. No statistically significant differences in edge preference were observed (H(3) = 1.65, P=.64, ε^2^ = 0.036).*

Nicotine caffeine co-treatment groups similarly did not have any statistically significant differences in edge preference (H(3) = 1.23, P=.75, ε^2^ = 0.009) between the treatment groups (n = 36 for each) of 100mg/L caffeine (91.66% [73.04–99.24]), 100mg/L caffeine + 0.93mg/L nicotine (95.11% [64.42–99.92]), 100mg/L caffeine + 1.85mg/L nicotine (85.67% [53.5–97.72]), and 100mg/L caffeine + 2.31mg/L nicotine (93.43% [76.58–98.65]). There also was no significant change in overall activity (H(3) = 0.58, P=.90, ε^2^ = 0.004).

*Figure 4.* *Percent of time spent near edge of well compared across 100mg/L caffeine, 100mg/L caffeine + 0.93mg/L nicotine, 100mg/L caffeine + 1.85mg/L nicotine, and 100mg/L caffeine + 2.31mg/L nicotine treatment groups (n=36 for each). Boxes show median and interquartile range, with individual datapoints overlaid. No statistically significant edge preference differences were observed (H(3) = 1.23, P=.75, ε^2^ = 0.009).*

# DISCUSSION

Although meclizine produced a statistically significant increase in thigmotaxis at 0.5 mg/L, it also caused a statistically significant decrease in activity. To analyze this difference, an activity-matched subgroup analysis was attempted, but only n=5 of 24 meclizine-treated larvae fell within the control activity interquartile range, indicating the magnitude of locomotor suppression. Therefore, it cannot be determined if the increase in edge preference at 0.5 mg/L reflects a true anxiogenic effect or is a byproduct of reduced activity. Additionally, 100 mg/L caffeine combined with 0.5 mg/L meclizine resulted in larvae mortality during extended observation, further limiting the ability to attribute the increase in thigmotaxis at this dose to an anxiogenic mechanism as opposed to a general toxic response.

The nicotine-alone experiment used n=12 larvae per group, which cannot reliably detect small-to-moderate effects in zebrafish behavior, so the absence of a significant effect at this sample size should be interpreted as inconclusive. Nicotine failed to reduce zebrafish edge-hugging behavior at any tested concentration, while meclizine produced an unexpected dose and stimulus specific response. Nicotine acts on nicotinic acetylcholine receptors as a stimulant, activating the sympathetic nervous system. Although nicotine has been seen to have a biphasic dose-response curve (anxiolytic at low doses while anxiogenic at high doses), its failure to act as an anxiolytic agent could be attributed to its state-dependent nature. Caffeine pre-exposure established a baseline excitatory drive that overrode the narrow therapeutic window of low-dose nicotine. Meclizine showed a different pattern, with 0.25 mg/L meclizine causing a significant increase in edge preference during the visual lines period when combined with caffeine. No similar increase was observed during first hour thigmotaxis and was not attributable to a baseline difference in motor activity. Sedation during the visual lines period could be a confounding factor, so this result should be treated as exploratory. These findings raise the possibility of an interaction between H1 receptor blockade and caffeine’s anxiogenic effects, which should be further analyzed in future studies. Together with the nicotine results, the data suggests that a GABAergic intervention like diazepam is required for a reliable rescue of caffeine-induced thigmotaxis. Future studies on other GABAergic interventions would be well suited to further analyze the relationship between such drugs and caffeine.
